# TRPA1 Integrates Nociceptive and Immune Signaling in *Hydra vulgaris*

**DOI:** 10.3390/ijms27104609

**Published:** 2026-05-21

**Authors:** Veronica D’Ezio, Valentina Malafoglia, Valeria Russo, Sara Ilari, Riccardo Proietti, Carolina Muscoli, Valentina Cianfanelli, Federica Spani, Massimiliano Scalici, Tiziana Persichini, Marco Colasanti

**Affiliations:** 1 Department of Science, University ROMA TRE, I-00146 Rome, Italy; veronica.dezio@uniroma3.it (V.D.); valeria.russo10@gmail.com (V.R.); riccardo.proietti@uniroma3.it (R.P.); valentina.cianfanelli@uniroma3.it (V.C.); massimiliano.scalici@uniroma3.it (M.S.); tiziana.persichini@uniroma3.it (T.P.); 2 Laboratory of Physiology and Pharmacology of Pain, IRCCS San Raffaele Roma, I-00163 Rome, Italy; valentina.malafoglia@sanraffaele.it (V.M.); sara.ilari@sanraffaele.it (S.I.); 3 Department of Health Sciences, Institute of Research for Food Safety and Health (IRC-FSH), University “Magna Graecia” of Catanzaro, I-88100 Catanzaro, Italy; muscoli@unicz.it; 4 Department of Science and Bio-Technology, Università Campus Bio-Medico di Roma, I-00128 Rome, Italy; f.spani@unicampus.it

**Keywords:** NF-κB, nociceptive–immune coupling, Nrf2, oxidative stress signaling, TRPA1 channel evolution

## Abstract

Transient receptor potential ankyrin 1 (TRPA1) channels detect noxious cold and inflammatory mediators in mammals; yet their evolutionary origins and roles in neuro-immune integration remain unclear. Here, we investigated TRPA1 in *Hydra vulgaris*, an early metazoan with a simple nervous system, exposing polyps to noxious cold and *Pseudomonas aeruginosa* lysate. Using Western blotting, pharmacological modulation, and gene expression analyses, we demonstrated that TRPA1 mediates upregulation of nociceptive markers (*Nrf2*, *NOS*, *SOD*) and immune effectors (*NF-κB*, *NOS*, *periculin*, *hydramacin*). TRPA1 antagonism significantly reduced these responses, indicating its role as an amplifier of both nociceptive and innate immune signaling. These findings suggest that TRPA1-dependent coupling of nociceptive-like and immune responses is an ancient, conserved mechanism, providing insights into the molecular basis of integrated threat detection and offering potential avenues for targeting pain and inflammation-associated pathologies.

## 1. Introduction

Transient receptor potential (TRP) channels form a superfamily of non-selective cation channels that respond to various stimuli, including thermal, mechanical, and chemical signals, thereby facilitating physiological adaptation and pathological sensitization [1]. Among the approximately 70 TRP channels identified in metazoans, TRPA1 (transient receptor potential ankyrin 1) functions as a primary sensor of noxious cold temperatures (<15 °C) in mammals, triggering sensory neuron activation and protective withdrawal behaviors [2]. TRPA1-mediated nociceptive signaling engages a coordinated molecular cascade of stress-responsive markers conserved across metazoan evolution. Key markers include nuclear factor erythroid 2-related factor 2 (Nrf2), nitric oxide synthase (NOS), and superoxide dismutase (SOD), which together serve as functional markers of TRPA1-activated threat-detection cascades involving oxidative stress and nociceptive-like responses [3,4,5]. Nrf2 acts as a rapid transcriptional sensor of oxidative stress, translocating to the nucleus within minutes to initiate the expression of cytoprotective genes [3]. NOS emerges as a prominent nociceptive effector enzyme, with robust upregulation occurring in both thermal and inflammatory pain contexts, serving a dual role as a pain mediator and immune signaling molecule [4]. SOD represents the sustained antioxidant buffering phase, accumulating as a delayed enzymatic defense that protects cells from sustained oxidative perturbation [5]. This temporal hierarchy—rapid Nrf2 sensing followed by delayed NOS and SOD accumulation—provides both immediate molecular alerting and long-term cellular protection, ensuring that acute stress does not exceed protective capacity before sustained antioxidant enzyme accumulation. Despite its widespread expression, TRPA1 thermosensitivity exhibits significant species-specific variations, suggesting that it has evolved as a polymodal sensor tailored to the ecological niches of diverse phyla [6].

Beyond thermosensation, TRPA1 participates in innate immune responses by detecting lipopolysaccharides (LPS) from Gram-negative bacteria, notably *Pseudomonas aeruginosa*, through pattern recognition receptor (PRR) coupling and reactive oxygen species (ROS) generation [7]. This dual role situates TRPA1 at the interface of neuro-immune homeostasis, where it operates simultaneously as a nociceptor and pathogen sensor. This modality aligns with the “Danger Model”, which posits that immune activation is primarily driven by danger signals or damage-associated molecular patterns (DAMPs) released by distressed cells rather than by recognition of foreignness alone [8]. Such recognition of the “damaged-self” represents a conserved biological principle observed across kingdoms, from plants to animals [9]. From an evolutionary perspective, the integration of the sensory and immune systems reflects an ancestral molecular convergence that enables discrimination between noxious stimuli and pathogens [10].

Clinical and preclinical evidence corroborates TRPA1 involvement in acute and chronic pain syndromes, as well as inflammatory diseases [11,12]. For example, TRPA1 expression in Schwann cells mediates neuroinflammation and mechanical hypersensitivity in a fibromyalgia model [13]. Accordingly, TRPA1 antagonists, such as HC-030031, have demonstrated efficacy in models of neuropathic and inflammatory pain [14]. On the other hand, activation of TRPA1 by endogenous agonists, including ROS, nitro-oxidative stress products, and bacterial LPS, induces NF-κB-dependent cytokine release, thereby amplifying nociceptive sensitization and immune responses [15]. This inflammatory amplification is critical in systemic pathologies; for instance, TRPA1 inhibition attenuates sepsis-induced organ damage by modulating the TLR4/NF-κB signaling pathway [16]. These findings suggest that TRPA1 may act either as an integrator of danger signals downstream of ancestral pattern recognition receptors or as a co-primary detector operating alongside co-receptors, such as TLR4 and TLR7, to coordinate multifaceted biological defense mechanisms [17,18,19] (for further details on TRPA1 signaling pathways, see Table S1 in the Supplementary Data).

A fundamental question in evolutionary neurobiology is the emergence of nociceptive systems in metazoan phyla. Comparative studies indicate that nociceptive-like behaviors and thermosensory responses are present in primitive organisms, although the interpretations remain cautious [20]. The evolutionary trajectory of these systems implies a shared origin of innate immunity and nociception in the early metazoan capacity to detect environmental threats [21]. Genetic analyses of *Hydra magnipapillata* have revealed an expansion of TRP channels, including approximately four TRPA, three TRPM, and five TRPVL channels, suggesting early diversification and fine-tuning of sensory functions within the cnidarian lineage [22]. However, functional evidence for TRPA1-mediated nociception and immune coupling in cnidarians remains lacking. Given their phylogenetic position, the freshwater polyp *Hydra vulgaris* [23] is an attractive model for exploring the evolutionary origin of TRPA1-dependent neuro-immune integration.

Converging nociceptive and immune signals into a unified framework may provide an obvious mechanistic advantage, as a damaged organism needs to detect the insult, amplify the signal, and initiate a response. A single polymodal TRPA1-like sensor, together with conserved transcriptional regulators, such as NF-κB and/or oxidative stress response elements, may enable rapid and coordinated activation of both neural and immune responses, thereby enhancing the organism ability to survive in environments with high microbial challenges.

In mammals, TRPA1 expression extends beyond sensory neurons to immune cells (neutrophils and macrophages), epithelial cells, and endothelial cells, highlighting its broad therapeutic relevance in pain, inflammation, and infection-associated diseases [24]. This extensive distribution enables TRPA1 to coordinate diverse physiological processes in the body. Previous studies have established TRPA1 as a molecular bridge between pattern recognition receptors (e.g., TLR4) and transcriptional immune mobilization via NF-κB, particularly in response to bacterial LPS [7]. Notably, the NF-κB protein in *Hydra vulgaris* exhibits a simplified structure containing only the Rel homology domain (RHD), indicating that complex immune regulatory mechanisms involving ankyrin domains evolved subsequently while preserving fundamental antimicrobial peptide control functions [25].

Genetic investigations in *H. magnipapillata* have identified two transmembrane TLR-like proteins, Hydra Toll-receptor-related 1 (HyTRR-1) and Hydra leucine-rich repeat receptor 2 (HyLRR-2), which exhibit canonical pattern recognition architectures [26,27]. HyTRR-1 contains an intracellular Toll/Interleukin-1 receptor (TIR) domain, characteristic of vertebrate TLRs, whereas HyLRR-2 possesses extracellular leucine-rich repeats (LRRs), typical of TLR recognition domains. Functionally, HyLRR-2 recognizes microbe-associated molecular patterns at the cell surface, whereas HyTRR-1 initiates intracellular signaling through MyD88-dependent cascades, culminating in NF-κB activation [26,27]. Silencing experiments have confirmed that both proteins are essential for antimicrobial peptide production, including hydramacin-1 and periculin-1, establishing Toll-like receptor-mediated immunity as an ancestral defense mechanism in cnidarians [25].

Building on this rationale, the present study aimed to (1) investigate TRPA1 role in *H. vulgaris* responses to noxious cold, characterizing its involvement in nociceptive-like pathways, and (2) determine whether TRPA1 acts as an amplifying node in immune responses to bacterial challenge, focusing on NF-κB-dependent antimicrobial peptide expression. We hypothesized that TRPA1 mediates a dual-mode function by transducing both acute thermosensation and amplifying inflammatory signaling.

## 2. Results

We investigated the function of TRPA1 in *H. vulgaris* to explore the potential primordial mechanisms underlying nociceptive–immune coupling. Our experimental approach integrated molecular and pharmacological techniques to establish mechanistic causality using TRPA1-selective tools, such as the agonist glybenclamide (G0639) and the antagonist 2-(1,3-Dimethyl-2,6-dioxo-1,2,3,6-tetrahydro-7H-purin-7-yl)-N-(4-isopropyl-phenyl)acetamide (HC-030031), and quantitative gene expression analyses.

### 2.1. Detection of TRPA1-like Protein in H. vulgaris

**Figure 1:**
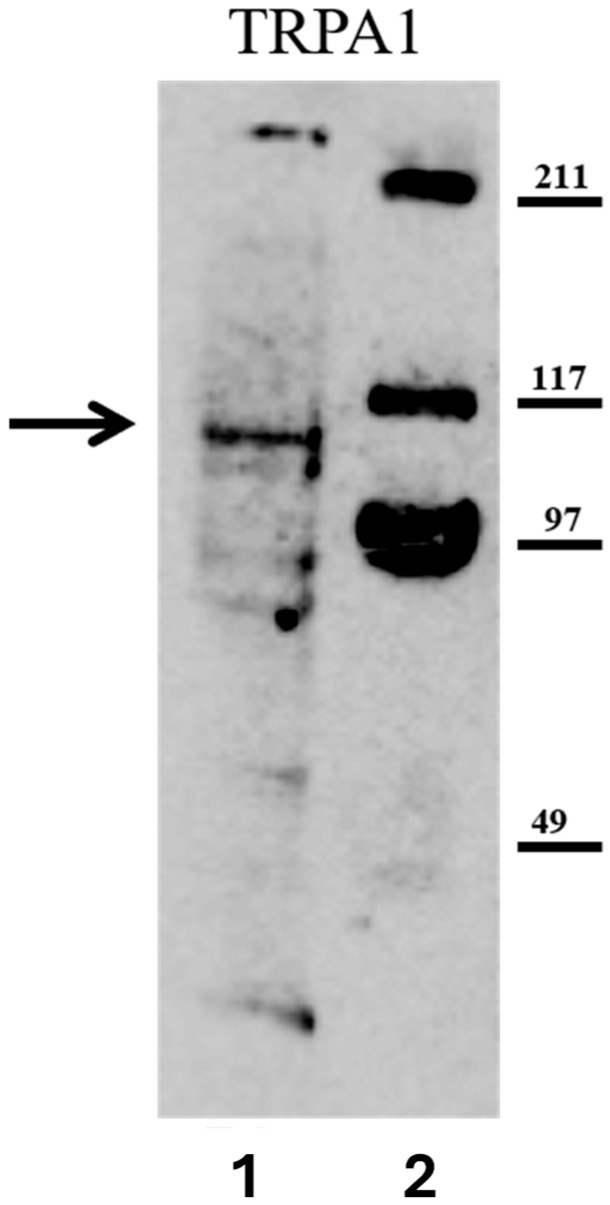
Detection of TRPA1-Like Protein in *Hydra vulgaris* by Western blotting. Lane 1: *H. vulgaris* whole polyp lysate (40 µg total protein) probed with anti-TRPA1 antibody. Lane 2: Molecular weight marker (prestained, 10–250 kDa). The arrow indicates the band at approximately 110 kDa, corresponding to the expected size of TRPA1 orthologs.

To establish the presence of TRPA1 in *H. vulgaris*, Western blot analysis was performed on whole-polyp lysates. A single immunoreactive band at approximately 110 kDa was consistently detected after incubation with anti-TRPA1 antibody (Figure 1). This molecular weight corresponds to the expected size of TRPA1 orthologs identified in other organisms and predicted by genomic analysis [22]. This finding establishes that *H. vulgaris* expresses a TRPA1-like protein, providing a molecular foundation for subsequent functional studies.

### 2.2. TRPA1 Agonist-Induced Gene Expression of Nociceptive Markers

**Figure 2:**
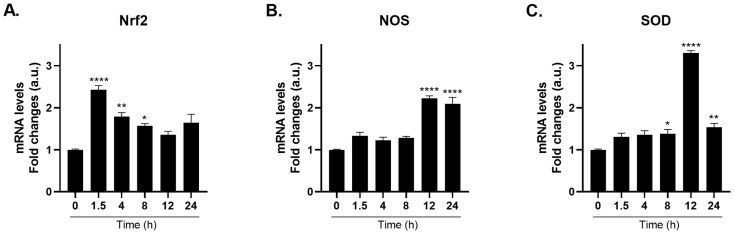
Selective TRPA1 Agonist Induces Time-Dependent Gene Expression of Nociceptive Markers in *Hydra vulgaris*. mRNA levels of *Nrf2* (**A**), *NOS* (**B**), and *SOD* (**C**) were measured by real-time PCR in *H. vulgaris* treated with 2 μM Glybenclamide (G0639) for the indicated times. Results were normalized to *β-actin* using the ΔΔCt method and expressed as mean fold change ± SEM (*n* = 9). Statistical significance: * *p* < 0.05, ** *p* < 0.01 and **** *p* < 0.0001 vs. 0 h control. Abbreviations: NOS (Nitric Oxide Synthase); Nrf2 (Nuclear factor erythroid 2-related factor 2); SOD (Superoxide dismutase).

To confirm that TRPA1 activation triggers nociceptive-associated gene expression, polyps were treated with the selective TRPA1 agonist Glybenclamide G0639 [14]. Treatment with 2 µM G0639 induced a time-dependent upregulation of nociceptive marker genes [3,4,5]. Specifically, Nrf2 mRNA levels increased approximately 2.4-fold at 1.5 h (Figure 2A), *NOS* mRNA levels increased approximately 2.2-fold at 12 h post-treatment (Figure 2B), and *SOD* mRNA levels increased approximately 3.3-fold at 12 h (Figure 2C) compared to the controls (for more details, see Table S2 in the Supplementary Data).

### 2.3. TRPA1 Antagonist Blocks Agonist-Induced Gene Expression of Nociceptive Markers

**Figure 3:**
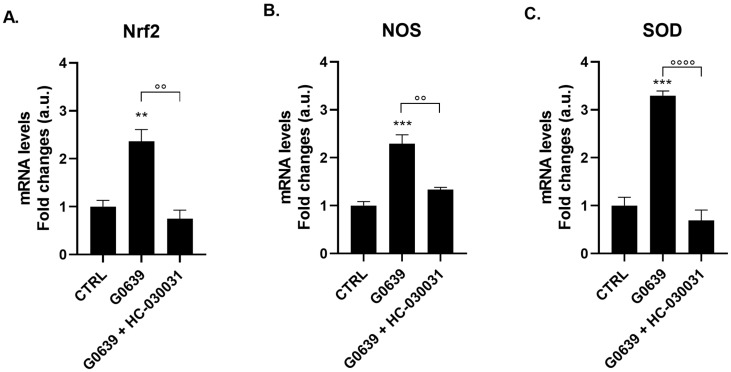
TRPA1 Antagonist (HC-030031) Blocks Agonist-Induced Gene Expression. mRNA levels of *Nrf2* (**A**), *NOS* (**B**), and *SOD* (**C**) were measured by real-time PCR in *H. vulgaris* pre-incubated for 2 h with the selective TRPA1 antagonist HC-030031 (0.1 µg/mL) and then treated with 2 μM Glybenclamide (G0639). Data were normalized to *β-actin* using the ΔΔCt method and expressed as mean fold change ± SEM (*n* = 9). Statistical significance: ** *p* < 0.01 and *** *p* < 0.001 vs. CTRL; and °° *p* < 0.01 and °°°° *p* < 0.0001 vs. G0639 agonist alone. Abbreviations: NOS (Nitric Oxide Synthase); Nrf2 (Nuclear factor erythroid 2-related factor 2); SOD (Superoxide dismutase).

To establish that G0639-induced gene expression is specifically mediated by TRPA1 and not through off-target pharmacology, polyps were pre-incubated with the selective TRPA1 antagonist HC-030031 (0.1 µg/mL, 2 h) before G0639 exposure. Pre-treatment with HC-030031 substantially blocked agonist-induced gene expression of all three nociceptive markers. Specifically, HC-030031 inhibited G0639-induced *Nrf2* expression from 2.36 ± 0.25 to 0.75 ± 0.18 (*p* < 0.01; Figure 3A), *NOS* expression from 2.29 ± 0.19 to 1.33 ± 0.05 (*p* < 0.01; Figure 3B), and *SOD* expression from 3.29 ± 0.10 to 0.69 ± 0.22 (*p* < 0.0001; Figure 3C). This inhibition pattern indicates that TRPA1 plays a substantial role in transducing this response. Although G0639 and HC-030031 are employed as selective TRPA1 modulators based on extensive mammalian literature [14,24], the possibility of off-target effects on other TRP channels in the expanded *Hydra* repertoire [22] cannot be entirely ruled out. Future studies employing genetic knockdown or CRISPR-based approaches in *Hydra* would provide definitive evidence regarding the specific TRP channels involved.

### 2.4. Behavioral Responses to Cold Shock

**Figure 4:**
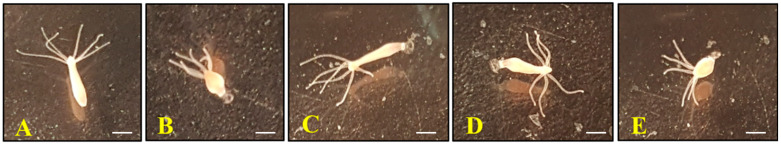
Behavioral Responses to Acute Noxious Cold Stress in *Hydra vulgaris*. Polyps subjected to noxious cold stress (4 °C for 1 min) exhibit acute functional impairment, demonstrating a behavioral phenotype consistent with a nociceptive-like stress response. (**A**) Baseline at 17 °C: polyp displays open tentacles and elongated body column characteristic of the physiological state. (**B**) Mechanical response at 17 °C: gentle needle contact elicits robust body contraction and tentacle retraction, confirming intact sensorimotor function. (**C**) Cold-shock phenotype (4 °C, 1 min): Polyps display profound functional impairment, including loss of contraction response, severely reduced tentacle reactivity, and diminished substratum adhesion. (**D**) Partial recovery phase: After 5 min of rewarming to 17 °C, polyps exhibited slowed movements with partial restoration of contractile capacity. (**E**) Complete recovery: After 10 min of rewarming at 17 °C, polyps fully restored baseline contractility, tentacle responsiveness, and normal body morphology. Magnification: 32×. Representative images from *n* = 15 polyps per condition across three independent biological replicates are shown. Scale bar 1 mm.

Prior to molecular analysis, we characterized the behavioral phenotype of cold-exposed polyps to confirm the presence of acute, noxious stress. Under normal physiological conditions (17 °C; Figure 4A), polyps responded robustly to mechanical stimulation (gentle needle contact) with characteristic body and tentacle contractions, indicating intact motor function (Figure 4B). Upon exposure to acute cold shock (4 °C for 1 min), the polyps exhibited pronounced behavioral changes, including reduced tentacle reactivity, diminished body contraction capacity, and temporary loss of substratum adhesion (Figure 4C). Quantitative analysis demonstrated that polyps at baseline (17 °C) maintained mean scores of approximately 2.8–2.9 across all behavioral parameters. Immediately following 1 min cold shock at 4 °C, these scores collapsed to 0.3–0.4 (all *p* < 0.0001), reflecting profound functional impairment. Critically, these cold-induced functional impairments were rapidly reversible after rewarming. Polyps returned to 17 °C for 5 min displayed slowed movements with partial recovery of contractility (Figure 4D), and scores returned to approximately 1.5–1.7. Full recovery of baseline behavioral responses was achieved after 10 min of rewarming (Figure 4E), with polyps fully restoring baseline behavior with scores of 2.7–2.8 (no significant difference from baseline). This rapid and complete reversibility, coupled with the absence of permanent morphological damage, confirms that 4 °C exposure induces acute nociceptive-like stress rather than generalized cellular toxicity. The temporal relationship between behavioral dysfunction and subsequent molecular responses suggests that cold stress-triggered nociceptive signaling initiates protective responses similar to those in mammalian nociception.

### 2.5. Cold Shock-Induced Gene Expression of Nociceptive Markers

**Figure 5:**
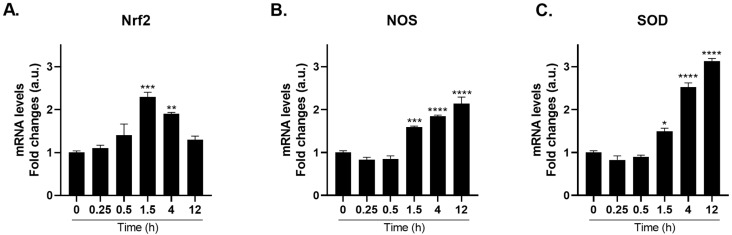
Cold Shock Induces Time-Dependent Gene Expression of Nociceptive Markers in *Hydra vulgaris*. mRNA levels of *Nrf2* (**A**), *NOS* (**B**), and *SOD* (**C**) were measured by real-time PCR in *H. vulgaris* shocked at 4 °C for 1 min before returning to 17 °C for the indicated times. Results were normalized to *β-actin* using the ΔΔCt method and expressed as mean fold change ± SEM (*n* = 9). Statistical significance: * *p* < 0.05, ** *p* < 0.01, *** *p* < 0.001 and **** *p* < 0.0001 vs. 0 h control. Abbreviations: NOS (Nitric Oxide Synthase); Nrf2 (Nuclear factor erythroid 2-related factor 2); SOD (Superoxide dismutase).

Polyps exposed to a single 1 min cold shock at 4 °C exhibited a time-dependent change in gene expression of nociceptive markers [3,4,5]. Among others, Nrf2 is a critical oxidative stress-responsive transcription factor that rapidly translocates to the nucleus upon sensing reactive oxygen species and initiates the transcription of downstream cytoprotective genes. *Nrf2* mRNA expression peaked at 1.5 h (2.3-fold) and returned to baseline by 12 h (Figure 5A).

The second phase, spanning 1.5–12 h, reflects delayed enzymatic amplification. While *Nrf2* peaked early, both *NOS* and *SOD* mRNA levels accumulated more slowly, reaching maximal induction at 12 h post-cold exposure. In particular, *NOS* mRNA levels increased approximately 2.1-fold at 12 h (Figure 5B), whereas *SOD* mRNA levels exhibited the most pronounced response, increasing approximately 3.1-fold at 12 h (Figure 5C) compared to the controls (for more details, see Table S3 in the Supplementary Data).

This temporal pattern suggests an early, transient activation of the transcriptional regulator Nrf2 as an initial “danger alert” phase in which the cell senses oxidative perturbation, followed by a progressive buildup of downstream antioxidant and nitrosative defense enzymes NOS and SOD. Consistently, these time-response data demonstrate that TRPA1 agonist produces gene expression kinetics that closely parallel those induced by the agonist G0639, suggesting that TRPA1 activation, whether thermal or chemical, engages a common downstream signaling cascade.

### 2.6. TRPA1 Antagonist Prevents Cold-Shock Induced Gene Expression of Nociceptive Markers

**Figure 6:**
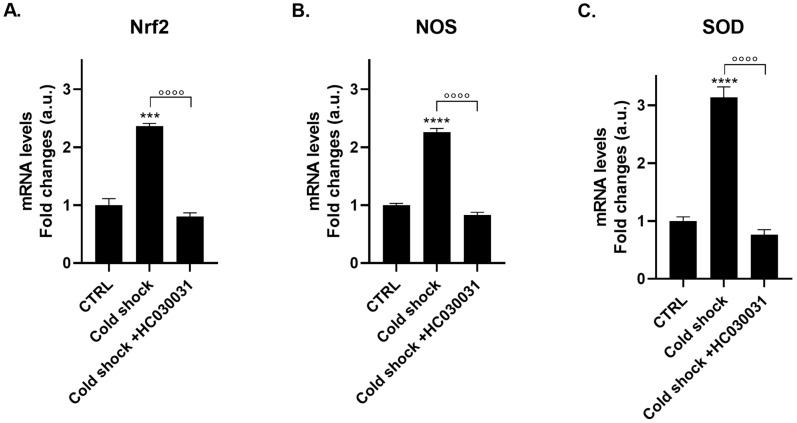
TRPA1 Antagonist (HC-030031) Blocks Cold-Induced Gene Expression. mRNA levels of *Nrf2* (**A**), *NOS* (**B**), and *SOD* (**C**) were measured by real-time PCR in *H. vulgaris* pre-incubated for 2 h with the selective TRPA1 antagonist HC-030031 (0.1 µg/mL) and then shocked at 4 °C for 1 min before returning to 17 °C. Data were normalized to *β-actin* using the ΔΔCt method and expressed as mean fold change ± SEM (*n* = 9). Statistical significance: *** *p* < 0.001 and **** *p* < 0.0001 vs. CTRL; and °°°° *p* < 0.0001 vs. cold shock alone. Abbreviations: NOS (Nitric Oxide Synthase); Nrf2 (Nuclear factor erythroid 2-related factor 2); SOD (Superoxide dismutase).

To establish the causal role of TRPA1 in mediating cold-induced gene expression, polyps were pre-treated with HC-030031 before exposure to cold shock. The selective TRPA1 antagonist substantially reduced all three nociceptive marker genes: *Nrf2* mRNA expression was reduced from 2.36 ± 0.04 to 0.80 ± 0.07 (*p* < 0.0001; Figure 6A), *NOS* mRNA expression from 2.3 ± 0.06 to 0.83 ± 0.05 (*p* < 0.0001; Figure 6B), and *SOD* expression from 3.13 ± 0.18 to 0.77 ± 0.08 (*p* < 0.0001; Figure 6C). Notably, the TRPA1-dependent component was remarkably consistent across all three markers, indicating that the cold-induced response is mediated by TRPA1 activation. However, parallel thermal sensing mechanisms cannot be excluded, consistent with the expanded TRP channel repertoire documented in the Hydra genome [22], which includes multiple TRPM and TRPV orthologs that may also contribute to cold sensing.

### 2.7. PA14 Bacterial Lysate-Induced Immune Response via TRPA1-Coupled Nociceptive Signaling

To investigate whether TRPA1 couples nociceptive and innate immune responses, polyps were treated with cell-free lysates from *Pseudomonas aeruginosa* PA14 (20 µL/mL).

Notably, the transcription factor NF-κB mediates transcriptional immune mobilization in response to TRPA1-dependent bacterial LPS induction [7]. PA14 lysate contains multiple PAMPs and virulence factors, including LPS, that represent a realistic bacterial immune challenge. Future studies using isogenic PA14 mutants or purified PAMPs would clarify which specific patterns engage TRPA1.

**Figure 7:**
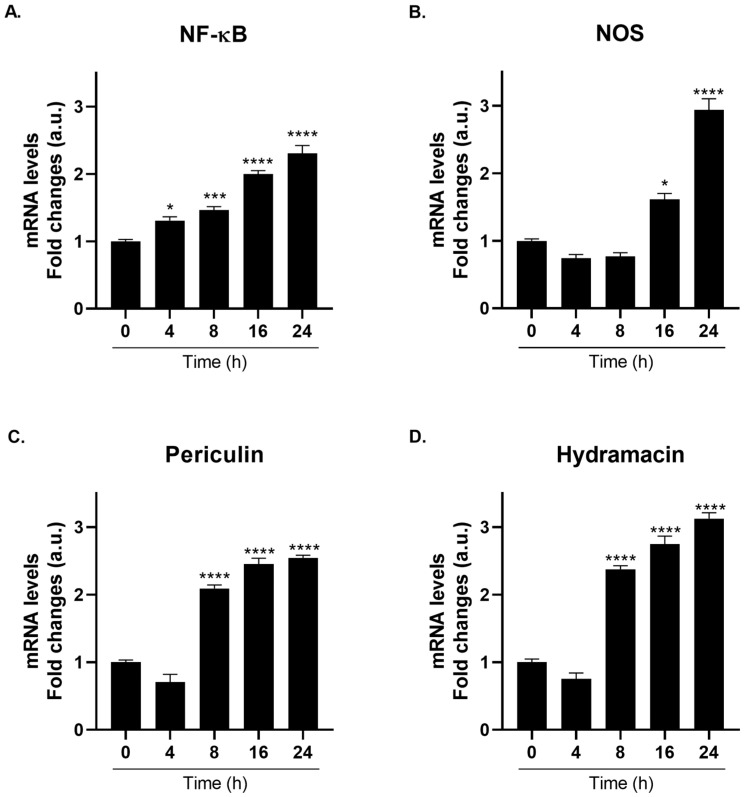
PA14 Bacterial Lysate Activates Integrated NF-κB-Dependent Immune Cascade in *Hydra vulgaris*. mRNA levels of *NF-κB* (**A**), *NOS* (**B**), *periculin* (**C**), and *hydramacin* (**D**) were measured by real-time PCR in *H. vulgaris* treated with *Pseudomonas aeruginosa* PA14 cell lysate (20 µL/mL) for the indicated times. Results were normalized to *β-actin* using the ΔΔCt method and expressed as mean fold change ± SEM (*n* = 9). Statistical significance: * *p* < 0.05, *** *p* < 0.001 and **** *p* < 0.0001 vs. 0 h control. Abbreviations: NF-κB (Nuclear Factor-κB); NOS (Nitric Oxide Synthase).

Here, we demonstrated that PA14 lysate induced a robust, time-dependent upregulation of *NF-κB* and downstream NF-κB-dependent immune effectors over a 24 h exposure window. Particularly, *NF-κB* showed a progressive time-dependent induction, reaching approximately 2.3-fold upregulation at 24 h (Figure 7A). *NOS* expression demonstrated particularly robust induction in the immune context, reaching approximately 3-fold upregulation at 24 h (Figure 7B), surpassing even *NF-κB* induction and establishing *NOS* as the most prominently elevated immune marker. *NOS* kinetics showed progressive induction, establishing a linear increase in NO production. The significant upregulation of *NOS* within bacterial immune contexts and nociceptive systems underscores the multifaceted role of NO as a coordinator of immune, stress, and regenerative processes [28,29,30,31].

Following *NF-κB* and *NOS* activation, the expression of NF-κB-dependent antimicrobial peptides, *periculin* and *hydramacin*, was robustly upregulated at 24 h. *Periculin* reached approximately 2.5-fold induction (Figure 7C), while *hydramacin* reached the highest absolute fold-change observed in immune experiments, approximately 3.1-fold (Figure 7D) compared to the controls (for more details, see Table S4 in the Supplementary Data). Both peptides exhibited progressive kinetics, with peak expression at 24 h, consistent with NF-κB-dependent transcriptional activation. These antimicrobial peptides represent the primary effector arsenal of *Hydra* [32,33], and their robust induction establishes a complete immune cascade from pattern recognition to transcriptional mobilization and antimicrobial effector production.

### 2.8. TRPA1 Antagonist Prevents PA14 Bacterial Lysate-Induced Gene Expression of Immune Markers

**Figure 8:**
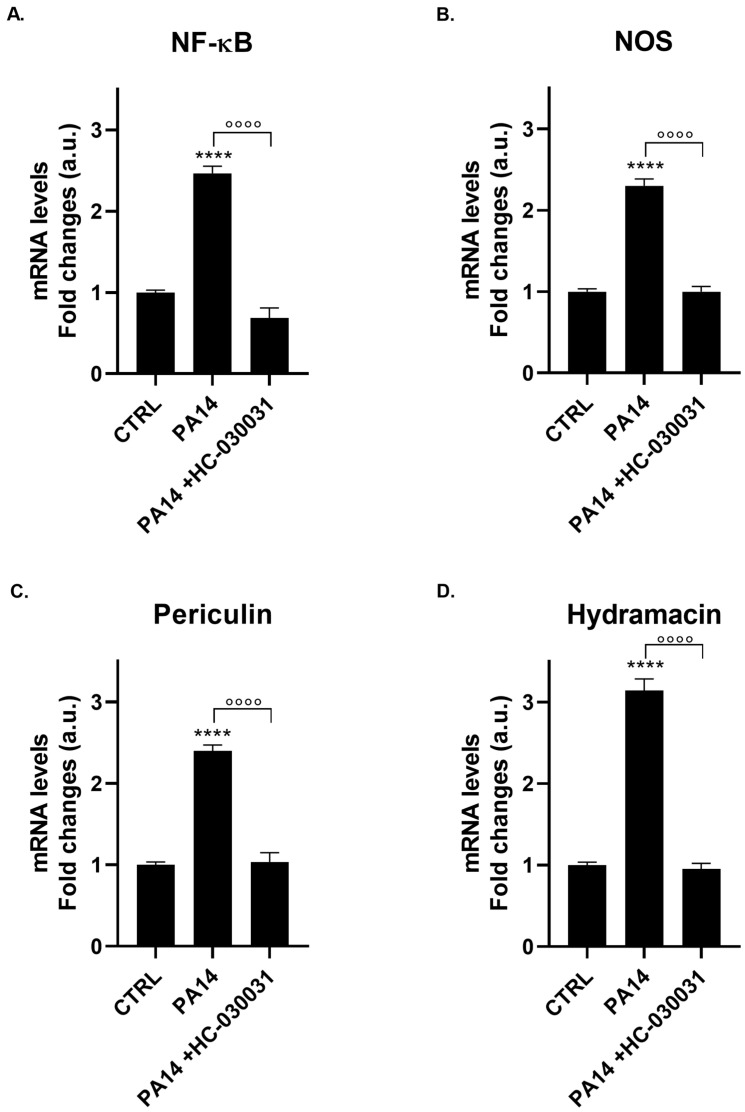
TRPA1 Antagonist (HC-030031) Blocks PA14 Bacterial Lysate-Induced Gene Expression. mRNA levels of *NF-κB* (**A**), *NOS* (**B**), *periculin* (**C**), and *hydramacin* (**D**) were measured by real-time PCR in *H. vulgaris* pre-incubated for 2 h with the selective TRPA1 antagonist HC-030031 (0.1 µg/mL) and then treated with *Pseudomonas aeruginosa* PA14 cell lysate (20 µL/mL). Data were normalized to *β-actin* using the ΔΔCt method and expressed as mean fold change ± SEM (*n* = 9). Statistical significance: **** *p* < 0.0001 vs. CTRL; and °°°° *p* < 0.0001 vs. PA14 treatment alone. Abbreviations: NF-κB (Nuclear Factor-κB); NOS (Nitric Oxide Synthase).

To establish that TRPA1 mediates the PA14-induced immune response, polyps were pre-incubated with HC-030031 (0.1 µg/mL, 2 h) before exposure to the PA14 lysate (20 µL/mL). The antagonist produced remarkable inhibition across all immune markers at the 24 h peak, specifically *NF-κB* gene expression from 2.47 ± 0.09 to 0.69 ± 0.12 (*p* < 0.0001; Figure 8A), *NOS* from 2.30 ± 0.08 to 1.00 ± 0.06 (*p* < 0.0001; Figure 8B), *periculin* from 2.4 ± 0.07 to 1.03 ± 0.11 (*p* < 0.0001; Figure 8C), and *hydramacin* from 3.14 ± 0.03 to 0.95 ± 0.07 (*p* < 0.0001; Figure 8D).

This consistent TRPA1-dependent inhibition across functionally distinct markers (transcription factors, signaling enzymes, and two independent antimicrobial peptides) indicates that TRPA1 acts as a critical amplification node within the PA14-induced immune cascade. The inhibition pattern mirrors that observed in cold shock responses, suggesting a conserved role for TRPA1 as a “danger-associated molecular amplifier” that enhances but does not exclusively control nociceptive or immune responses. Statistical analysis confirmed highly significant effects of HC-030031 antagonism (all *p* < 0.0001), validating the mechanistic importance of TRPA1 in bacterial sensing. However, parallel sensing mechanisms cannot be ruled out, consistent with the expanded TRP channel repertoire documented in the Hydra genome [22], which includes multiple TRPM and TRPV orthologs.

## 3. Discussion

This study aimed to clarify TRPA1 role as a molecular integrator of threat detection functions by examining its kinetic and mechanistic responses to two distinct stressors: noxious cold and bacterial challenge. Prior research has demonstrated that TRPM3 mediates the heat-induced upregulation of *Nrf2*, *NOS*, and *SOD* in *H. vulgaris* [5], indicating the involvement of TRP channels in oxidative stress pathways. Building on this foundation, the present study characterized TRPA1-specific mechanisms in both nociceptive and immune contexts.

### 3.1. Temporal Organization and Context-Dependent TRPA1 Activation

Temporal kinetics between cold shock and PA14-immune activation diverged despite shared TRPA1 involvement. Cold exposure induced a biphasic response: rapid *Nrf2* upregulation peaked at 1.5 h, followed by delayed *NOS* and *SOD* accumulation at 12 h. This temporal hierarchy reveals an elegant stress defense architecture in which rapid transcription factor mobilization serves as an immediate “alarm signal”, that triggers a slower accumulation of enzymatic buffering capacity. This organization permits responsiveness without allowing transient stressors to overwhelm cellular defenses [34].

In contrast, PA14 bacterial lysate triggered a multi-step cascade with immune markers—*NF-κB*, *NOS*, *periculin*, and *hydramacin*—peaking synchronously at 24 h. This pattern mirrors mammalian innate immune mechanisms, in which NOS activation drives antimicrobial NO production and inflammation [30].

These findings suggest that TRPA1 employs context-responsive activation mechanisms: cold triggers fast Nrf2 sensing and sustained enzymatic accumulation, whereas inflammatory signals proceed via delayed NF-κB-dependent responses. This distinction reflects the different propagation speeds through direct channel sensing versus TLR-mediated pathways, enabling TRPA1 to function as a context-responsive molecular switch.

### 3.2. TRPA1 and Nitric Oxide as Integrators of Nociceptive–Immune Signaling

A striking finding in both contexts was the prominence of NOS pathway upregulation. In bacterial-challenged polyps, *NOS* induction (3-fold at 24 h) exceeded *NF-κB* activation (2.3-fold), establishing NO production as the dominant transcriptional response to PAMP challenges. This prominence mirrors mammalian mechanisms, wherein NO serves a dual role as both an antimicrobial effector and a stress-responsive signaling molecule [31].

The coupling of NOS induction to TRPA1 activation in both nociceptive and immune contexts suggests that NO is a fundamental molecular currency of TRPA1-mediated danger signaling in early metazoans. The evolutionary conservation of NO pathways reflects the ancient integration of pain and immune functions. It should be pointed out, moreover, that NO has been implicated in the hydra feeding response, which represents a primitive olfactory-like behavior in a multicellular organism [35,36].

The TRPA1-dependence of both cold and immune responses may function as a critical amplification node within multi-receptor detection systems. In cold sensing, for example, TRPA1 may cooperate with TRPM and TRPV orthologs, each of which potentially detects distinct biophysical aspects of thermal stress. In bacterial immune sensing, TRPA1 can operate in association with TLR4/TLR7-MyD88 signaling, where ROS generated by initial TLR activation oxidatively modify TRPA1 ankyrin repeat cysteine residues, enabling channel opening and amplifying downstream NF-κB cascades [7,37,38].

This amplification model confers both evolutionary and mechanistic advantages: it allows for sensitivity tuning, couples danger sensing to the cellular redox state, and provides a molecular basis for the convergence of diverse danger signals on TRPA1. The observation that TRPA1-dependent inhibition occurs across various functional markers, including transcription factors, signaling enzymes, and antimicrobial peptides, supports the role of TRPA1 as an amplification node that enhances but does not exclusively control nociceptive and immune responses.

### 3.3. Ancestral Integration of Nociceptive and Immune Functions

The TRPA1-mediated coupling of nociceptive and immune signaling in *H. vulgaris* suggests that this integration may represent an ancestral metazoan trait that predates the emergence of bilaterian animals over 600 million years ago. This coupling indicates that early animals evolved mechanisms for coordinated responses to threats, whereby tissue damage or pathogen exposure activates the same molecular pathways (TRPA1-NF-κB-NOS) to initiate both protective nociceptive-like responses and antimicrobial defenses [28,39]. Such integration likely conferred a survival advantage by enabling rapid and coordinated responses to environmental threats without the need for separate sensory systems. The presence of TLR pathway components (HyTRR-1, HyLRR-2, and NF-κB) in *Hydra* [28], together with the amplification role of TRPA1, supports an evolutionary model in which pattern recognition receptor (PRR)-mediated immune sensing is functionally integrated with temperature and redox sensing via TRPA1-dependent ROS amplification. This integration would have allowed ancestral organisms to mount rapid and coordinated responses to environmental threats. By converging nociceptive and immune functions within a single polymodal sensor, organisms can achieve efficient threat detection and response, reducing the energetic costs associated with maintaining separate sensory and immune systems.

### 3.4. TRPA1 Architectural Models and Evolutionary Conservation

Despite extensive research, the precise architectural role of TRPA1 within the broader immune hierarchy remains to be fully elucidated. One plausible model posits that TRPA1 functions as a secondary amplification layer downstream of ancestral pattern recognition systems. In this scenario, HyTRR-1 and HyLRR-2 mediate initial PAMP recognition and TLR-dependent priming, whereas TRPA1 activation triggered by TLR4-induced ROS generation selectively amplifies the transcriptional immune response [28,40]. This hierarchical mechanism enables precise calibration of immune responses and represents an evolutionarily efficient strategy for mounting proportional defenses against threats.

Alternatively, TRPA1 may function as a co-equal primary sensor operating in parallel with ancestral PRRs, facilitating redundant threat detection and robust immune mobilization in response to pathogens [17,41]. This modular organization provides evolutionary flexibility, as the disruption of any single pathway does not eliminate immune capability, and parallel mechanisms maintain responsiveness. Both models predict the observed TRPA1-dependent contribution and align with the evidence of multifunctional sensory neuron integration in immune contexts [17,41].

The evolutionary conservation of this TRPA1-NF-κB-NOS axis over the past 600+ million years suggests that this integrated threat-detection mechanism represents an ancient and fundamental solution for coordinating nociceptive and immune responses. If similar pathways prove operational in mammalian infection and pain models [42], such findings could provide mechanistic support for the evolutionary hypothesis that pain and immunity coupling serves coordinated defense functions. Whether clinical observations in pain care strategies and rehabilitation programs might corroborate this evolutionary perspective remains an open and intriguing question for future investigations.

In summary, TRPA1 functions as a unified “danger-alarm” that activates different biological responses in different contexts. Cold stress activates nociceptive-like responses, facilitating withdrawal, alongside Nrf2-driven antioxidant gene expression for cell survival. Bacterial challenges trigger immune activation, leading to the release of pro-inflammatory mediators and antimicrobial peptides. The convergence of nociceptive, immune, and metabolic functions within TRPA1 reflects evolutionary parsimony: a unified “master alarm” is more efficient than separate detection pathways, illustrating the optimization of molecular resources under evolutionary pressure. This ancient system integrates danger detection, signal amplification, and coordinated responses, offering an evolutionary solution for rapid and proportional threat responses in early metazoans.

## 4. Materials and Methods

This section describes the materials, organisms, experimental protocols, and analytical methods employed to investigate TRPA1-mediated nociceptive and immune signaling in *H. vulgaris*.

### 4.1. Reagents and Biological Materials

All chemical reagents and biological materials were sourced from established commercial suppliers to ensure reproducibility and quality. *H. vulgaris* polyps, kindly provided by dr. Claudia Tortiglione (NanoBiomolecular Group, CNR, Pozzuoli, Italy), were maintained in Hydra culture medium consisted of CaCl_2_ (1 mM) and NaHCO_3_ (1 mM) prepared in deionized water. *Pseudomonas aeruginosa* PA14 (ATCC BAA-1244) was purchased from the American Type Culture Collection (ATTC, Manassas, VA, USA) and cultured according to established protocols.

For pharmacological studies, the selective TRPA1 agonist glybenclamide (G0639) and the antagonist 2-(1,3-Dimethyl-2,6-dioxo-1,2,3,6-tetrahydro-7H-purin-7-yl)-N-(4-isopropylphenyl)acetamide (HC-030031) (both from Sigma-Aldrich, St. Louis, MO, USA) [14] were prepared as stock solutions in dimethyl sulfoxide (DMSO). Working concentrations were freshly prepared in Hydra medium immediately before use. The final DMSO concentration did not exceed 0.1% (*v*/*v*).

Molecular biology reagents included TRIzol (Life Technologies, Carlsbad, CA, USA) for RNA extraction, GoTaq 2-Step RT-qPCR System (Promega, Madison, WI, USA) for reverse transcription and PCR amplification, and SYBR Green PCR Master Mix (Biorad, Hercules, CA, USA) for real-time quantitative reactions. Gene-specific primers for *Nrf2*, *NOS*, *SOD*, *NFκB*, *periculin*, *hydramacin*, and the reference gene *β-actin* were designed using Primer3Plus software v1.0.8 and synthesized by Integrated DNA Technologies (IDT, Coralville, IA, USA). The primer sequences are listed in Table 1.

For protein analysis, we used an anti-TRPA1 primary antibody (rabbit polyclonal, 1:100 dilution; Alomone Labs, Jerusalem, Israel) and an anti-rabbit horseradish peroxidase-conjugated secondary antibody (1:3000 dilution; Amersham, GE Healthcare Europe, Milan, Italy) for Western blotting. Enhanced chemiluminescence (ECL) reagent (Amersham), Laemmli sample buffer and nitrocellulose membranes (Bio-Rad Laboratories, Milan, Italy) were used for protein immunodetection.

Additional reagents and buffer systems included Tryptic Soy Broth (TSB) Tris-buffered saline (TBS), Tween-20, bovine serum albumin (BSA), Triton X-100, sodium dodecyl sulfate (SDS), β-mercaptoethanol, chloroform, isopropanol, ethanol, and protease inhibitor cocktails (all from Sigma-Aldrich). All reagents were of analytical or molecular biology grade.

### 4.2. Organism Maintenance and Experimental Design

We cultured *H. vulgaris* polyps asexually at 17 ± 1 °C in Hydra medium under a 16 h light/8 h dark photoperiod to maintain physiological consistency. Polyps were fed once a week with newly hatched *Artemia salina* nauplii and transferred to fresh medium every 48 h to ensure adequate nutrition and environmental conditions for their growth. Only visually healthy polyps at 2–4 weeks after budding and without morphological anomalies were selected for the experiments.

For all experiments, polyps were randomly assigned to treatment groups. We selected sample sizes (*n* = 15 polyps per group across three independent biological replicates, with three technical replicates per sample, yielding *n* = 9 measurements per group) to provide adequate statistical power for detecting significant changes in gene expression. All procedures were performed in accordance with institutional guidelines.

### 4.3. Behavioral Assessment and Morphological Analysis

We assessed the morphological integrity and behavioral responses of polyps before and after experimental treatments using light microscopy (Leica DM IL, Leica Microsystems, Wetzlar, Germany) at 32× magnification. We monitored three behavioral variables: (1) tentacle retraction and responsiveness to gentle mechanical stimulation, (2) body elongation and column contraction dynamics, and (3) adhesion to the substratum. Each parameter was scored on a 0–3 scale, where 0 indicated complete loss of function and 3 indicated normal baseline responsiveness. Representative images were captured at baseline and key time points to document morphological changes and functional recovery. Statistical comparisons were made using one-way ANOVA with Bonferroni post hoc test (*n* = 15 polyps per condition across three independent biological replicates).

### 4.4. Cold Shock Exposure

To simulate acute noxious cold stress, we acclimated the polyps to 17 °C and then rapidly transferred them to 4 °C Hydra medium for 1 min before returning them to 17 °C. We collected samples at specific time points post-rewarming: 0 (immediately), 0.25, 0.5, 1.5, 4, and 12 h. At each time point, we first assessed behavioral responses using light microscopy, then rapidly froze the polyps in liquid nitrogen and stored them at −80 °C until nucleic acid extraction.

### 4.5. Bacterial Lysate Preparation

We cultured *Pseudomonas aeruginosa* PA14 overnight (16–18 h) in TSB at 37 °C with vigorous agitation. Bacterial cells were harvested by centrifugation at 5000× *g* for 15 min at 4 °C, washed thrice with sterile 0.9% NaCl, and resuspended to an OD_600_ of 0.5. The cells were then lysed by sonication (three consecutive 5 min pulses at 40% amplitude). The resulting cell-free lysate was centrifuged at 10,000× *g* for 10 min at 4 °C, filtered through sterile 0.45 µm PVDF membrane filters to ensure sterilization, and stored at −20 °C in aliquots for use within four weeks.

For treatment, *H. vulgaris* polyps were exposed to 20 µL of PA14 lysate per well in 48-well tissue culture plates containing 1 mL Hydra medium. The control polyps received equivalent volumes of sterile saline. Polyps were incubated under standard conditions, and samples were collected at 0 (immediate), 4, 8, 16, and 24 h after lysate addition.

### 4.6. Pharmacological Modulation of TRPA1

Polyps were incubated with 2 µM TRPA1 agonist G0639 for 24 h under standard culture conditions, and samples were collected at 0, 1.5, 4, 8, 12, and 24 h.

For the antagonist experiments, polyps were pre-incubated with HC-030031 at a concentration of 0.1 µg/mL for 2 h before exposure to (i) the agonist G0639 (2 µM), (ii) PA14 lysate (20 µL/mL) for 24 h, or (iii) CS (4 °C) for 1 min before returning them to 17 °C for 12 h. The polyps were maintained in the antagonist-containing medium throughout the experimental period.

All pharmacological treatments included vehicle controls (DMSO at ≤0.1% *v*/*v* final concentration). Stock solutions were freshly prepared on experimental days and stored on ice to maintain chemical stability.

### 4.7. RNA Extraction and Real-Time Quantitative Polymerase Chain Reaction (RT-qPCR)

Total RNA was extracted from frozen *H. vulgaris* polyps (*n* = 9 replicates per group) using TRIzol reagent, following the manufacturer’s instructions. Briefly, individual polyps were homogenized in 1 mL TRIzol, incubated for 5 min at room temperature, and then phase-separated with chloroform (200 µL per mL TRIzol) by centrifugation at 12,000× *g* for 15 min at 4 °C. The aqueous phase was precipitated with isopropanol (500 µL per mL of original TRIzol), washed twice with 75% ethanol, and resuspended in 50 µL DEPC-treated water. We verified RNA integrity using NanoDrop 2000 spectrophotometry (Thermo Fisher Scientific, Wilmington, DE, USA), confirming optical density ratios of 260/280 nm between 1.8 and 2.0 and 260/230 nm greater than 2.0.

Complementary DNA (cDNA) was synthesized using the GoTaq 2-Step RT-qPCR System (Promega Corporation, Madison, WI, USA), according to the manufacturer’s instructions. Briefly, we combined total RNA (1 µg) with oligo(dT) primers and heated the mixture at 70 °C for 5 min. Following cooling to 4 °C, reverse transcriptase, dNTP mix, and reaction buffer were added, and thermal cycling was performed at 25 °C for 5 min (annealing), 42 °C for 60 min (synthesis), and 70 °C for 15 min (inactivation). The resulting cDNA was diluted 1:10 in nuclease-free water and stored at −20 °C until use.

Real-Time Quantitative PCR (qPCR) was performed using the Agilent AriaMx Real-Time PCR System (Agilent Technologies, Santa Clara, CA, USA) with SYBR Green supermix (Bio-Rad Italia, Milan, Italy). Each 15 µL reaction contained 3 µL of diluted cDNA template, 7.5 µL of 2× SYBR Green PCR Master Mix, 0.3 µL of each forward and reverse primer (25 µM stock), and 3.9 µL of nuclease-free water. All amplicons were designed to be approximately 100 bp long. The thermal cycling program consisted of initial polymerase activation at 95 °C for 15 s, followed by 45 amplification cycles (95 °C for 15 s, 60 °C for 60 s, 72 °C for 20 s) and melt curve analysis (65–95 °C at 0.5 °C increments per 5 s) to verify single-product amplification. All reactions were performed in technical triplicates, with no-template negative controls. We obtained Ct values from AriaMx software v1.7.1 and calculated relative mRNA quantification using the ΔΔCt method, with *β-actin* as the reference gene. Results are expressed as mean fold-change ± standard error of the mean (SEM) relative to untreated control polyps at *t* = 0.

### 4.8. Western Blot Analysis

*H. vulgaris* polyps (*n* = 9 replicates per group) were homogenized in ice-cold lysis buffer (50 mM Tris-HCl pH 7.4, 150 mM NaCl, 1% Triton X-100, 0.1% SDS, and a complete protease inhibitor cocktail) using a motorized pestle. Homogenates were incubated for 30 min at 4 °C with periodic vortexing and centrifuged at 14,000× *g* for 15 min at 4 °C to pellet cellular debris. We stored the resulting supernatant at −80 °C and determined the protein concentration using the Pierce BCA Protein Assay Kit (Thermo Fisher Scientific, Rockford, IL, USA), with bovine serum albumin as the standard.

We mixed protein lysates (15 µg total protein per lane) with 2× Laemmli sample buffer supplemented with 5% β-mercaptoethanol, heated at 95 °C for 5 min, and loaded onto 7.5% polyacrylamide gels. Electrophoresis was performed at 150 V for approximately 1.5 h. Proteins were transferred to 0.2 µm nitrocellulose membranes at a constant voltage of 20 V for 30 min. We verified the transfer efficiency using Ponceau S staining.

Membranes were blocked for 2 h at room temperature in 1% BSA/TBST and then incubated overnight at 4 °C with anti-TRPA1 primary antibody (1:100 dilution in 1% BSA/TBST). After four 5 min washes in TBST, the membranes were incubated for 1 h at room temperature with horseradish peroxidase-conjugated anti-rabbit secondary antibody (1:3000 dilution). Following four additional 5 min washes, immunoreactive bands were detected using ECL substrate, and chemiluminescent signals were captured by digital imaging using a ChemiDoc Gel Documentation System (Bio-Rad Laboratories, Hercules, CA, USA).

### 4.9. Statistical Analysis

We performed statistical analyses using GraphPad Prism v8.0 and verified all results using the R statistical computing environment (v. 4.2.0). Data are presented as mean ± standard error of the mean (SEM) from *n* = 9 technical measurements per group across three independent biological replicates.

For normally distributed data, we applied one-way analysis of variance (ANOVA) with Bonferroni’s multiple comparison post hoc test. For two-group comparisons, we employed either an unpaired Student’s *t*-test (parametric) or the Mann–Whitney U test (non-parametric), as appropriate. All statistical tests were two-tailed (α = 0.05). We considered *p*-values less than 0.01 as highly significant (*p* < 0.01), values between 0.01 and 0.05 as significantly different (*p* < 0.05), and values *p* > 0.05 as not statistically significant (ns).

Generative AI was used in this paper to assist in bibliographic research, as well as in the stylistic editing and linguistic refinement of the text. All scientific findings and interpretations remain the original work of the authors.

## 5. Conclusions

Our findings in *H. vulgaris* suggest that TRPA1 serves as a crucial molecular integrator and amplifier of nociceptive and immune signaling that has been maintained for over 600 million years of metazoan evolution. The conservation of these responses implies that TRPA1-mediated “danger” sensing is a primordial feature of animal life [8,20]. TRPA1 functional conservation between *Hydra* and mammals suggests that pain and immunity coupling is an ancient evolutionary solution predating the vertebrate adaptive immune system. Rather than viewing pain as a sophisticated neural feature of complex organisms, our data raise questions about whether primitive nociceptive-like mechanisms arose at the base of the metazoan lineage as an integrated threat detection and defense component.

Despite their significance, these results have some important limitations. Future studies should directly investigate the structural and biochemical mechanisms underlying the gating of TRPA1 in ancestral species, dissect the precise pathways of ROS-TRPA1 interactions, and perform more comprehensive comparative genomic analyses in other metazoan phyla. It is also interesting to verify whether similar pathways are operational in mammalian infection and pain models to determine their clinical relevance.

Collectively, our data tentatively support the hypothesis that TRPA1 operates as a primordial ‘danger alarm’ that coordinates nociceptive and immune functions at the base of the metazoan tree, while acknowledging that further validation is required. If confirmed, this framework could inform therapeutic strategies targeting multi-system TRPA1-dependent pathologies and advance our understanding of how ancient organisms evolved to integrate solutions for threat detection and defense.

## Figures and Tables

**Table 1:** RT-qPCR Primer Sequences for Gene Expression Analysis.

Gene	Forward (5′→3′)	Reverse (5′→3′)
*Nrf2*	CTAGTAGAGTCATTATCTCC	AAACTTGAATCTGACCTCTG
*NOS*	TATCAAGCAGCAGGTGTGAC	TACAGATCCAGAAAGCGGAG
*SOD*	TCAGTTGTGGGATTATTCAGGTG	CAAACACCGGGAAATGCTGGA
*NF-κB*	ATAATTTCCCGCAACCCGGC	AAAATCAGGAATCGCCGGAG
*Periculin*	TGGGTACAAACCCAAGAAGG	CTATATAACCAGCTCTGGGC
*Hydramacin*	GATGCACGAAATGCAGTCAG	TTAGTAACAGATGCAAGCCC
*β-actin*	TCCTTGTATGCTTCTGGTCG	ATAATGGCATGGGCAAGAGC

## Data Availability

The original contributions presented in this study are included in the article/Supplementary Materials. Further inquiries can be directed at the corresponding author.

## Supplementary Materials

The following supporting information can be downloaded at: https://www.mdpi.com/article/10.3390/ijms27104609/s1, refs. [43,44,45] have been cited in Supplementary Materials.

## Abbreviations

The following abbreviations are used in this manuscript:

CS	Cold Stress
DAMPs	Damage-associated molecular patterns
DMSO	Dimethyl sulfoxide
HyLRR-2	Hydra leucine-rich repeat receptor 2
HyTRR-1	Hydra Toll-receptor-related 1
LPS	Lipopolysaccharide
LRRs	Leucine-rich repeats
NF-κB	Nuclear Factor-κB
NOS	Nitric oxide synthase
Nrf2	Nuclear factor erythroid 2-related factor 2
PA14	*Pseudomonas aeruginosa*
PAMPs	Pathogen-Associated Molecular Patterns
PRR	Pattern recognition receptor
RHD	Rel homology domain
ROS	Reactive oxygen species
RT-qPCR	Real-Time Quantitative Polymerase Chain Reaction
SOD	Superoxide dismutase
TIR	Toll/Interleukin-1 receptor
TLR	Toll-like receptor
TRP	Transient receptor potential
TRPA1	Transient receptor potential ankyrin 1

## References

[1] Ramsey I.S., Delling M., Clapham D.E. (2006). An Introduction to Trp Channels. Annu. Rev. Physiol..

[2] Story G.M., Peier A.M., Reeve A.J., Eid S.R., Mosbacher J., Hricik T.R., Earley T.J., Hergarden A.C., Andersson D.A., Hwang S.W. (2003). ANKTM1, a TRP-like Channel Expressed in Nociceptive Neurons, Is Activated by Cold Temperatures. Cell.

[3] Petrikonis K., Bernatoniene J., Kopustinskiene D.M., Casale R., Davinelli S., Saso L. (2024). The Antinociceptive Role of Nrf2 in Neuropathic Pain: From Mechanisms to Clinical Perspectives. Pharmaceutics.

[4] Luo Z.D., Cizkova D. (2000). The Role of Nitric Oxide in Nociception. Curr. Rev. Pain.

[5] Malafoglia V., Traversetti L., Del Grosso F., Scalici M., Lauro F., Russo V., Persichini T., Salvemini D., Mollace V., Fini M. (2016). Transient Receptor Potential Melastatin-3 (TRPM3) Mediates Nociceptive-Like Responses in *Hydra vulgaris*. PLoS ONE.

[6] Tominaga M., Iwata M. (2025). TRPA1 and Thermosensitivity. J. Physiol. Sci..

[7] Meseguer V., Alpizar Y.A., Luis E., Tajada S., Denlinger B., Fajardo O., Manenschijn J.-A., Fernández-Peña C., Talavera A., Kichko T. (2014). TRPA1 Channels Mediate Acute Neurogenic Inflammation and Pain Produced by Bacterial Endotoxins. Nat. Commun..

[8] Matzinger P. (2002). The Danger Model: A Renewed Sense of Self. Science.

[9] Heil M., Land W.G. (2014). Danger Signals-Damaged-Self Recognition across the Tree of Life. Front. Plant Sci..

[10] Kraus A., Buckley K.M., Salinas I. (2021). Sensing the World and Its Dangers: An Evolutionary Perspective in Neuroimmunology. eLife.

[11] Nassini R., Materazzi S., Benemei S., Geppetti P., Nilius B., Gudermann T., Jahn R., Lill R., Offermanns S., Petersen O.H. (2014). The TRPA1 Channel in Inflammatory and Neuropathic Pain and Migraine. Reviews of Physiology, Biochemistry and Pharmacology.

[12] Qi Y., Gong H., Shen Z., Wu L., Xu Z., Shi N., Lin K., Tian M., Xu Z., Li X. (2025). TRPM8 and TRPA1 Ideal Targets for Treating Cold-Induced Pain. Eur. J. Med. Chem..

[13] Brum E.S., Fialho M.F.P., De Araújo D.S.M., Landini L., Marini M., De Logu F., Nassini R., Oliveira S.M. (2026). Schwann Cell TRPA1, a Proalgesic Ion Channel, Mediates Neuroinflammation and Fibromyalgia-Associated Behaviours in Mice. J. Neurochem..

[14] Babes A., Fischer M.J.M., Filipovic M., Engel M.A., Flonta M.-L., Reeh P.W. (2013). The Anti-Diabetic Drug Glibenclamide Is an Agonist of the Transient Receptor Potential Ankyrin 1 (TRPA1) Ion Channel. Eur. J. Pharmacol..

[15] Bautista D.M., Movahed P., Hinman A., Axelsson H.E., Sterner O., Högestätt E.D., Julius D., Jordt S.-E., Zygmunt P.M. (2005). Pungent Products from Garlic Activate the Sensory Ion Channel TRPA1. Proc. Natl. Acad. Sci. USA.

[16] Fırat S., Çakır M., Aydın A., Bircan B., Şekerci G., Tekin S. (2025). The Ameliorative Effect of Inhibiting Transient Receptor Potential Ankyrin 1 on Sepsis-Induced Kidney Injury via the Toll-like Receptor 4/Nuclear Factor-Kappa B Pathway. Eur. J. Pharmacol..

[17] Donnelly C.R., Chen O., Ji R.-R. (2020). How Do Sensory Neurons Sense Danger Signals?. Trends Neurosci..

[18] Michot B., Casey S.M., Lee C.S., Erdogan O., Basu H., Chiu I., Gibbs J.L. (2023). Lipopolysaccharide-Induced TRPA1 Upregulation in Trigeminal Neurons Is Dependent on TLR4 and Vesicular Exocytosis. J. Neurosci..

[19] Park C.-K., Xu Z.-Z., Berta T., Han Q., Chen G., Liu X.-J., Ji R.-R. (2014). Extracellular MicroRNAs Activate Nociceptor Neurons to Elicit Pain via TLR7 and TRPA1. Neuron.

[20] Walters E.T. (2025). From Nociception in Aneural Animals to Human Suffering: Toward a Comparative Biology of Pain. J. Exp. Biol..

[21] Buchmann K. (2014). Evolution of Innate Immunity: Clues from Invertebrates via Fish to Mammals. Front. Immunol..

[22] Peng G., Shi X., Kadowaki T. (2015). Evolution of TRP Channels Inferred by Their Classification in Diverse Animal Species. Mol. Phylogenet. Evol..

[23] Chapman J.A., Kirkness E.F., Simakov O., Hampson S.E., Mitros T., Weinmaier T., Rattei T., Balasubramanian P.G., Borman J., Busam D. (2010). The Dynamic Genome of Hydra. Nature.

[24] Tekulapally K.R., Lee J.Y., Kim D.S., Rahman M.M., Park C.-K., Kim Y.H. (2024). Dual Role of Transient Receptor Potential Ankyrin 1 in Respiratory and Gastrointestinal Physiology: From Molecular Mechanisms to Therapeutic Targets. Front. Physiol..

[25] Glass B.H., Abraham T., Siggers T., Davies S.W., Gilmore T.D. (2026). NF-κB: A Diverse and Multifunctional Transcription Factor in Holozoans. Mol. Biol. Evol..

[26] Bosch T.C.G., Augustin R., Anton-Erxleben F., Fraune S., Hemmrich G., Zill H., Rosenstiel P., Jacobs G., Schreiber S., Leippe M. (2009). Uncovering the Evolutionary History of Innate Immunity: The Simple Metazoan Hydra Uses Epithelial Cells for Host Defence. Dev. Comp. Immunol..

[27] Hemmrich G., Miller D.J., Bosch T.C.G. (2007). The Evolution of Immunity: A Low-Life Perspective. Trends Immunol..

[28] Augustin R., Fraune S., Bosch T.C.G. (2010). How Hydra Senses and Destroys Microbes. Semin. Immunol..

[29] Colasanti M., Mazzone V., Mancinelli L., Leone S., Venturini G. (2009). Involvement of Nitric Oxide in the Head Regeneration of *Hydra vulgaris*. Nitric Oxide Biol. Chem..

[30] Colasanti M., Persichini T., Venturini G. (2010). Nitric Oxide Pathway in Lower Metazoans. Nitric Oxide Biol. Chem..

[31] Colasanti M., Suzuki H. (2000). The Dual Personality of NO. Trends Pharmacol. Sci..

[32] Fraune S., Augustin R., Anton-Erxleben F., Wittlieb J., Gelhaus C., Klimovich V.B., Samoilovich M.P., Bosch T.C.G. (2010). In an Early Branching Metazoan, Bacterial Colonization of the Embryo Is Controlled by Maternal Antimicrobial Peptides. Proc. Natl. Acad. Sci. USA.

[33] Jung S., Dingley A.J., Augustin R., Anton-Erxleben F., Stanisak M., Gelhaus C., Gutsmann T., Hammer M.U., Podschun R., Bonvin A.M.J.J. (2009). Hydramacin-1, Structure and Antibacterial Activity of a Protein from the Basal Metazoan Hydra. J. Biol. Chem..

[34] Itoh K., Igarashi K., Hayashi N., Nishizawa M., Yamamoto M. (1995). Cloning and Characterization of a Novel Erythroid Cell-Derived CNC Family Transcription Factor Heterodimerizing with the Small Maf Family Proteins. Mol. Cell Biol..

[35] Colasanti M., Venturini G., Merante A., Musci G., Lauro G.M. (1997). Nitric Oxide Involvement in *Hydra vulgaris* Very Primitive Olfactory-like System. J. Neurosci..

[36] Colasanti M., Lauro G.M., Venturini G. (1995). NO in Hydra Feeding Response. Nature.

[37] Ko H.-K., Lin A.-H., Perng D.-W., Lee T.-S., Kou Y.R. (2020). Lung Epithelial TRPA1 Mediates Lipopolysaccharide-Induced Lung Inflammation in Bronchial Epithelial Cells and Mice. Front. Physiol..

[38] Parks T., Taylor-Clark T. (2019). Complete NEM Modification of Highly Reactive Cysteines Induces Full TRPA1 Activation. FASEB J..

[39] Franzenburg S., Fraune S., Künzel S., Baines J.F., Domazet-Lošo T., Bosch T.C.G. (2012). MyD88-Deficient *Hydra* Reveal an Ancient Function of TLR Signaling in Sensing Bacterial Colonizers. Proc. Natl. Acad. Sci. USA.

[40] Janeway C.A., Medzhitov R. (2002). Innate Immune Recognition. Annu. Rev. Immunol..

[41] Erdogan O., Hu X.-Q., Chiu I.M. (2025). Sensory Neurons on Guard: Roles in Pathogen Defense and Host Immunity. Curr. Opin. Immunol..

[42] Baraldi P.G., Preti D., Materazzi S., Geppetti P. (2010). Transient Receptor Potential Ankyrin 1 (TRPA1) Channel as Emerging Target for Novel Analgesics and Anti-Inflammatory Agents. J. Med. Chem..

[43] Aubdool A.A., Graepel R., Kodji X., Alawi K.M., Bodkin J.V., Srivastava S., Gentry C., Heads R., Grant A.D., Fernandes E.S. (2014). TRPA1 is essential for the vascular response to environmental cold exposure. Nat. Commun..

[44] Talavera K., Startek J.B., Alvarez-Collazo J., Boonen B., Alpizar Y.A., Sanchez A., Naert R., Nilius B. (2020). Mammalian Transient Receptor Potential TRPA1 Channels: From Structure to Disease. Physiol. Rev..

[45] Viana F. (2016). TRPA1 channels: Molecular sentinels of cellular stress and tissue damage. J. Physiol..

