# Supplementary material for: TRPA1 Integrates Nociceptive and Immune Signaling in Hydra vulgaris
Source: Int J Mol Sci. 2026 May 21;27(10):4609. doi: 10.3390/ijms27104609 (PMC13207855; doi:10.3390/ijms27104609)
Supplement: Supplementary file 1 [file ijms-27-04609-s001.zip › ijms-4310464-supplementary.pdf]

International Journal of *Molecular Sciences*

MDPI

Supplementary Materials

TRPA1 Integrates Nociceptive and Immune Signaling in *Hydra vulgaris*

Veronica D'Ezio [1,†], Valentina Malafoglia [2,†], Valeria Russo [1], Sara Ilari [2], Riccardo Proietti [1], Carolina Muscoli [3], Valentina Cianfanelli [1], Federica Spani [4], Massimiliano Scalici [1], Tiziana Persichini [1] and Marco Colasanti [1,*]

Table S1. TRPA1 signaling pathways [1-4][1]

Stimuli	Signaling Pathways	Molecular responses	Biological Effects	Organ / Cell Systems
Oxidative Stress & Electrophiles (ROS, AITC, 4-HNE)[2]	Activation of the antioxidant response via Nrf2/ARE pathway.	Upregulation of SOD1/SOD2; HO-1 induction.	Cytoprotection, ROS scavenging, protection against tissue damage.	Lung, Liver, Sensory Neurons.
Inflammatory/Bacterial (LPS, TNF-α, IL-1β)	Nuclear translocation of p65 (NF-κB); sensitization via PAR2.	Transcription of the *iNOS* gene.	Release of pro-inflammatory cytokines, neurogenic inflammation.	Macrophages, Bronchial cells, Gut.
Noxious Cold (Extreme cold <17°C)	Voltage-dependent gating; Ca2+ influx activating Nrf2 response.	Balance between NO and ROS scavenging.	Acute cold pain, cold allodynia, cold-triggered headache.	Sensory Neurons (C-fibers), Trigeminal Ganglia.
Endogenous Mediators (Bile acids, Prostaglandins)	GPCR sensitization (TGR5, PAR2); modulation by PIP2.	TRPA1 deletion (KO) reduces iNOS and inflammatory markers.	Pruritus (itch), visceral hypersensitivity (IBS), chronic pain.	Gastrointestinal Tract, Skin.
Tissue Damage & Ischemia	NO/cGMP Signaling: Interaction with NOS activity and CGRP release.	Modulation of constitutive nNOS/eNOS isoforms via Ca^{2+}.	Vasodilation, response to hypoxia, sensing metabolic stress.	Cardiovascular System, Trigeminal Nerve.
Environmental Pollutants (Smoke, Acrolein)	Ca^{2+}-dependent p38/JNK and NF-κB activation.	Upregulation of pro-inflammatory enzymes (iNOS, COX-2).	Chronic cough, airway hyperresponsiveness, lung fibrosis.	Respiratory System (Bronchial Epithelium).
Chemotherapeutics (Oxaliplatin, Paclitaxel)	Activation via ROS production and GSH depletion.	Linked to CIPN.	Burning pain, mechanical and cold hyperalgesia.	Peripheral Nervous System.
Redox Balance Crosstalk	Nrf2 acts as a negative regulator of NF-κB.	Regulation of the NO/ROS balance for repair.	Resolution of inflammation, neuroprotection, wound healing.	Central Nervous System, Immune System.

[1] This table focuses on some aspects of the biological role of TRPA1. This option imposes restrictions, and interested readers are invited to consult valuable papers on this topic. References used for the table are: [1] Aubdool et al. Nat Commun 2014, 5, 5732, doi:10.1038/ncomms6732. [2] Tekulapally et al. Front. Physiol. 2024, 15, 1413902, doi:10.3389/fphys.2024.1413902. [3] Talavera et al. Physiological Reviews 2020, 100, 725–803, doi:10.1152/physrev.00005.2019. [4] Viana et al. The Journal of Physiology 2016, 594, 4151–4169, doi:10.1113/JP270935.

[2] Abbreviations used in the table: 4-HNE: 4-Hydroxynonenal; AITC: Allyl Isothiocyanate; ARE: Antioxidant Response Element; Ca^{2+}: Calcium Ion; CGRP: Calcitonin Gene-Related Peptide; CIPN: Chemotherapy-Induced Peripheral Neuropathy; COX-2: Cyclooxygenase-2; eNOS: Endothelial Nitric Oxide Synthase; GSH: Glutathione; GPCR: G-Protein Coupled Receptor; HO-1: Heme Oxygenase-1; IBS: Irritable Bowel Syndrome; iNOS: Inducible Nitric Oxide Synthase; IL-1β: Interleukin-1β; JNK: c-Jun N-terminal Kinase; KO: Knockout; nNOS: Neuronal Nitric Oxide Synthase; NO: Nitric Oxide; p38: p38 Mitogen-Activated Protein Kinase; p65: Transcription factor p65 (RelA); PAR2: Protease-Activated Receptor 2; PIP2: Phosphatidylinositol 4,5-bisphosphate; SOD1: Superoxide Dismutase 1 (Cu/Zn-SOD); SOD2: Superoxide Dismutase 2 (Mn-SOD); TGR5: G protein-coupled bile acid receptor 1 (GPBAR1); TNF-α: Tumor Necrosis Factor.

Table S2. Raw qPCR Data: TRPA1 Agonist (G0639) Induces a Time-Dependent Gene Expression

Time (h)	*Nrf2* (Fold Change)	*NOS* (Fold Change)	*SOD* (Fold Change)
0[1]	1.00 ± 0.02	1.00 ± 0.01	1.00 ± 0.02
1.5	2.43 ± 0.10****	1.33 ± 0.08	1.31 ± 0.09
4	1.79 ± 0.10**	1.22 ± 0.08	1.36 ± 0.09
8	1.57 ± 0.05*	1.28 ± 0.04	1.39 ± 0.10*
12	1.36 ± 0.08	2.22 ± 0.06****	3.31 ± 0.05****
24	1.64 ± 0.20	2.09 ± 0.16****	1.54 ± 0.09**

[1] Control; *$p < 0.05$, ** $p < 0.01$ and **** $p < 0.0001$ vs. 0 h control. Data were expressed as mean fold change ± SEM

Table S3. Raw qPCR Data: Cold shock (4°C) Induces a Time-Dependent Gene Expression.

Time (h)	*Nrf2* (Fold Change)	*NOS* (Fold Change)	*SOD* (Fold Change)
0[1]	1.00 ± 0.04	1.00 ± 0.04	1.00 ± 0.04
0.25	1.10 ± 0.07	0.83 ± 0.09	0.83 ± 0.09
0.5	1.40 ± 0.26	0.85 ± 0.07	0.90 ± 0.04
1.5	2.30 ± 0.11***	1.59 ± 0.03***	1.49 ± 0.07*
4	1.90 ± 0.03**	1.84 ± 0.03****	2.52 ± 0.10****
12	1.30 ± 0.08	2.14 ± 0.15****	3.13 ± 0.06****

[1] Control; *$p < 0.05$, ** $p < 0.01$, *** $p < 0.001$ and **** $p < 0.0001$ vs. 0 h control. Data were expressed as mean fold change ± SEM

Table S4. Raw qPCR Data: PA14 Bacterial Lysate Induces a Time-Dependent Gene Expression.

Time (h)	*NF-κB* (Fold Change)	*NOS* (Fold Change)	*Periculin* (Fold Change)	*Hydramacin* (Fold Change)
0[1]	1.00 ± 0.03	1.00 ± 0.03	1.00 ± 0.03	1.00 ± 0.04
4	1.31 ± 0.05*	0.74 ± 0.05	0.71 ± 0.11	0.75 ± 0.09
8	1.50 ± 0.05***	0.77 ± 0.05	2.09 ± 0.05****	2.38 ± 0.05****
16	2.00 ± 0.05****	1.62 ± 0.08*	2.46 ± 0.08****	2.75 ± 0.11****
24	2.31 ± 0.12****	2.94 ± 0.16****	2.55 ± 0.04****	3.13 ± 0.09****

[1] Control; * $p < 0.05$, *** $p < 0.001$ and **** $p < 0.0001$ vs. 0 h control. Data were expressed as mean fold change ± SEM
